# Compliance of systematic reviews and meta-analyses in ophthalmology with the PRISMA statement: an AI-based assessment and longitudinal comparison with 2017 data

**DOI:** 10.1186/s12874-026-02825-0

**Published:** 2026-03-17

**Authors:** Seon Young Lee, Jae Seon Hong, Sang Hyeok Lee, Rajen Gupta

**Affiliations:** 1 Department of Ophthalmology, James Cook University Hospital, Middlesbrough, England; 2 Royal Victoria Infirmary, Newcastle upon Tyne, England; 3 North Tees University Hospital, Stockton-on-Tees, England; 4 Department of Ophthalmology, Royal Victoria Infirmary, Newcastle upon Tyne, England

**Keywords:** PRISMA statement, Systematic reivews, Meta-analysis, AI-based assessments, Reporting quality, Ophthalmology, Longitudinal comparison

## Abstract

**Background:**

Systematic reviews and meta-analyses are vital in evidence-based medicine, especially in ophthalmology, where the complexity of paired data can lead to reporting challenges. In 2017, we evaluated the adherence of ophthalmology-related systematic reviews and meta-analyses to the PRISMA (Preferred Reporting Items for Systematic reviews and Meta-Analyses) 2009 statement. This study revisits the issue with a focus on adherence to the updated PRISMA 2020 checklist, compares results with the 2017 study, and explores the potential of AI in evaluating compliance.

**Objective:**

The aim of this study is to evaluate the reporting quality of systematic reviews and meta-analyses published in major ophthalmology journals between 2020 and 2024, based on the PRISMA 2020 checklist, and to compare human and AI assessments of compliance.

**Methods:**

A total of 207 systematic reviews and meta-analyses published in 11 major ophthalmology journals were included in this study. Each article was independently assessed for adherence to the 2020 PRISMA checklist, first by two human reviewers, and subsequently by two distinct AI platforms (ChatGPT-4.0 and Gemini Pro 2.5). Compliance scores were calculated, and inter-observer agreement between human and AI evaluations was determined using Cohen’s kappa statistic. The Mann–Whitney U test was employed to compare these findings with those of a 2017 study.

**Results:**

The mean compliance score, as assessed by human reviewers, was 36.28 out of 42 points (86.37%), indicating a substantial improvement in adherence to the PRISMA checklist compared with the level reported in the 2017 study (*p* < 0.00001). Compliance scores generated by the AI platforms demonstrated a moderate level of agreement with human assessments (Cohen’s κ = 0.63 for ChatGPT, 0.54 for Gemini). Strong compliance was observed for background and rationale (items 3 and 4), selection criteria (items 5–10b), and limitations (items 23a–23c). Conversely, lower compliance was noted for risk of bias assessment (item 11), sensitivity analysis (items 13f and 20c), and research registration (items 24a–24c).

**Conclusions:**

This study demonstrates a marked improvement in the reporting quality of systematic reviews and meta-analyses in ophthalmology following adoption of the 2020 PRISMA statement. Nonetheless, persistent deficiencies remain, particularly in the reporting of bias, sensitivity analyses, and research registration. The application of AI models offers promising potential for enhancing the efficiency and effectiveness of reporting quality assessments; however, further refinement is required to ensure consistency and accuracy. Future iterations of the PRISMA guidelines should consider explicitly addressing the role of AI in research evaluation.

**Supplementary Information:**

The online version contains supplementary material available at 10.1186/s12874-026-02825-0.

## Background

The importance of systematic reviews and meta-analyses in the practice of evidence-based medicine cannot be overstated. In recent years, the volume of published medical literature has increased exponentially, rendering individual studies insufficient to provide the comprehensive context necessary for informed clinical decision-making [1, 2]. Consequently, it has become increasingly challenging for healthcare professionals to remain abreast of every new study. Systematic reviews and meta-analyses address this gap by synthesizing existing research, enabling clinicians to access the most current and reliable evidence in a concise and interpretable format [2, 3]. With the growing number of such reviews and their pivotal role in guiding practice, the accuracy, transparency, and rigor of their complete reporting have become more critical than ever. High-quality reporting ensures the reliability of conclusions, supports reproducibility, and ultimately enhances the translation of evidence into clinical care [1, 4].

In 2017, we conducted a study assessing the overall reporting quality of ophthalmology systematic reviews and meta-analyses, focusing on adherence to the Preferred Reporting Items for Systematic reviews and Meta-Analyses (PRISMA) 2009 checklist. During this work, we noted a unique challenge in ophthalmic research: data frequently pertain to a single individual but are represented as two separate entities (bilateral), such as right and left eye measurements. This results in the inclusion of paired data, alongside cases where only unilateral data are available. Such complexity introduces specific considerations for the unit of analysis, statistical processing, and interpretation of results, such as the need to define whether analyses are performed at the eye level or the patient level, to account for inter-eye correlation when bilateral data are included, to appropriately handle mixed unilateral and bilateral observations, and to ensure that the chosen statistical models and reported outcomes accurately reflect within-subject dependence. Consequently, reporting in ophthalmology studies is inherently more intricate than in many other disciplines. Inadequate reporting in these contexts can obscure study design and analytical methods, potentially leading to misinterpretation and inappropriate application in clinical practice. This underscores the importance of regularly evaluating the reporting quality of published literature in this field [5].

In 2020, the PRISMA statement was updated, providing a revised checklist aimed at improving the transparency and completeness of systematic reviews [6]. Given that five years have elapsed since this update, a follow-up study is warranted to reassess the reporting quality of more recent ophthalmology systematic reviews and meta-analyses. Such an investigation would not only provide an updated appraisal of current practices but also enable direct comparison with our 2017 findings to identify any changes or trends over time.

In parallel, studies from a few other medical specialties have applied the PRISMA 2020 statement to evaluate the reporting quality of systematic reviews and meta-analyses, demonstrating that suboptimal adherence to updated reporting standards is a widespread issue rather than one confined to a single discipline. Collectively, this growing body of meta-research situates the present study within a broader effort to assess the real-world uptake and impact of PRISMA 2020, rather than addressing a problem unique to ophthalmology alone [7–9].

One of the most prominent and rapidly evolving technological domains in recent years has been artificial intelligence (AI), which has seen expanding applications in medicine, including data analysis and processing, drug discovery, diagnosis, treatment planning, and scientific publishing. It is almost certain that AI will continue to play a pivotal role in medical research, presenting opportunities for its integration into a wide range of scholarly and clinical activities [10–12]. The PRISMA checklist remains a cornerstone tool for ensuring transparent and standardized reporting in systematic reviews and meta-analyses. Accordingly, evaluating the capacity of AI to assess publications based on the PRISMA checklist is critical for determining its current utility and exploring its potential future applications in enhancing the quality and efficiency of research reporting [13].

In this study, we aim to evaluate the reporting quality of articles published in leading ophthalmology journals by assessing each publication’s adherence to the PRISMA 2020 checklist and statement. Furthermore, we will compare these findings with the results of our 2017 study, thereby enabling an assessment of changes in reporting quality over time. In addition, we seek to compare the outcomes of human assessments of PRISMA compliance with those generated through AI-based evaluations, examining both the degree of concordance between the two approaches and the potential role of AI in supporting systematic review reporting quality assessments.

## Methods

This study has been registered with the Research Registry (Registration number: researchregistry11448, hyperlink: https://www.researchregistry.com/browse-the-registry/#home/registrationdetails/689bc03fde0c6802f2830c0e/). No modifications have been made to the manuscript as a result of the registration. Institutional review board approval was not required, as there were no human participants or animal experiments involved in this study.

### Inclusion criteria

The study inclusion criteria were as follows: systematic reviews and meta-analyses published in English between 2020 and 2024 in the top 10 ophthalmology journals ranked by impact score as of December 2024. The criteria used to define systematic reviews and meta-analyses were based on the Cochrane Library guideline; high-level, reliable summary of primary research on a specific healthcare question [14].

The definition of an ophthalmology journal in this study primarily followed the categorisation used for impact factor rankings. Considering that the Royal College of Ophthalmologists defines Ophthalmology as ‘a branch of medicine dealing with the diagnosis, treatment and prevention of diseases of the eye and visual system,’ [15] the impact factor–based journal categorisation was considered an appropriate proxy for identifying ophthalmology journals.

The exclusion criteria comprised all articles that did not meet the inclusion criteria. In addition, studies whose primary focus was not related to ophthalmology or eye/vision research were excluded.

### Search strategy

A systematic search was conducted in Ovid MEDLINE(R) ALL (1946 to May 1, 2025) and Embase (1974 to May 1, 2025). The search was designed to identify articles published between 1 January 2020 and 31 December 2024. In each database, the strategy combined controlled vocabulary (MeSH in MEDLINE and Emtree in Embase) and relevant free-text terms, and results were restricted to records indexed as systematic reviews and/or meta-analyses using the databases’ publication type and indexing filters.

To limit retrieval to the target journals, a journal name (source title) filter was applied within each database. Specifically, the search was restricted to records where the journal/source field matched one of the top 11 ophthalmology journals by impact factor as of December 2024: *Progress in Retinal and Eye Research*,* Ophthalmology*,* JAMA Ophthalmology*,* The Ocular Surface*,* Survey of Ophthalmology*,* Annual Review of Vision Science*,* Clinical and Experimental Ophthalmology*,* Contact Lens and Anterior Eye*,* American Journal of Ophthalmology*,* British Journal of Ophthalmology*,* and Asia-Pacific Journal of Ophthalmology.* While the original study protocol prespecified inclusion of the top 10 journals, 11 journals were ultimately included due to a tied impact factor ranking. To ensure methodological consistency and avoid arbitrary exclusion, both journals sharing the same rank were retained. In Ovid MEDLINE, this restriction was implemented by limiting to the journal name (JN) field; in Embase, the equivalent source title/journal field limit was used. This ensured that only articles published in the pre-specified journals were retrieved.

The search strategy was finalised and re-run on May 1, 2025 to minimise the risk of missing eligible articles, as publication metadata for articles released late in the calendar year may be updated after initial indexing. The detailed search strategy is provided in Appendix 1.

### Study selection process

After retrieval of the article list through the database search, a manual selection process was undertaken. Any duplicate records were removed. The remaining articles were independently screened by two researchers (JSH and SHL) based on title and abstract. Subsequently, full-text articles were retrieved and assessed for eligibility to complete the final selection process. Articles were excluded if they were not systematic reviews or meta-analyses, or if they were not directly related to ophthalmology. Umbrella reviews were not excluded, as their methodological characteristics are comparable to those of systematic reviews and meta-analyses.

Prior to the screening process, the two reviewers (JSH and SHL) received structured training on the definitions and methodological criteria of systematic reviews and meta-analyses to ensure a clear and consistent understanding of the study types being assessed. Screening was conducted only after consensus was reached regarding the operational definitions.

**Fig. 1:**
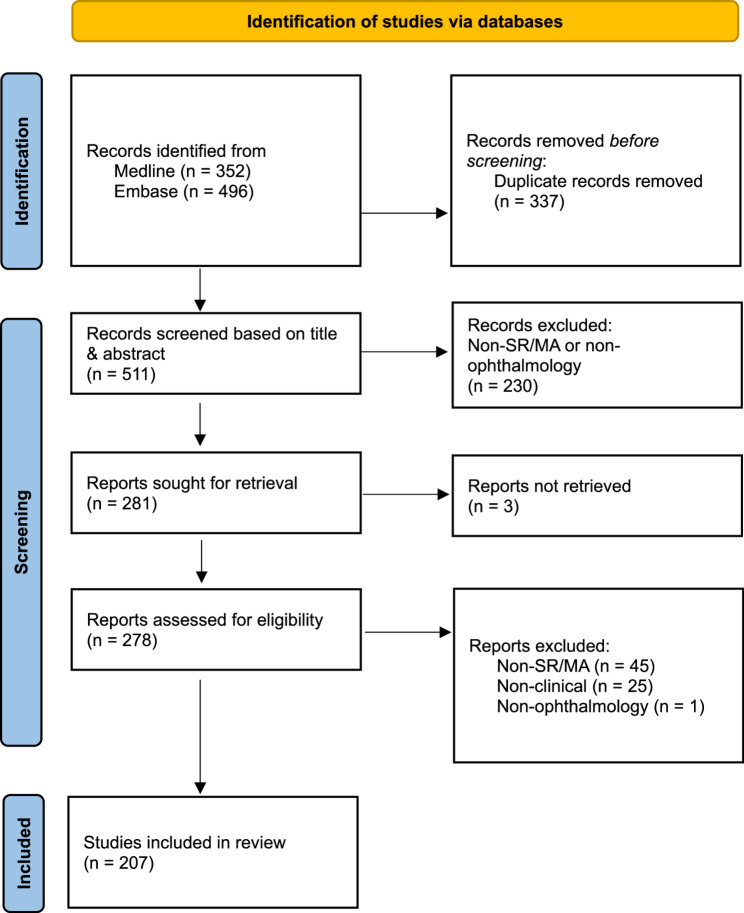
PRISMA Flow Diagram of the Study Selection Process Source: Page MJ, et al. BMJ 2021;372:n71. 10.1136/bmj.n71. This work is licensed under CC BY 4.0. To view a copy of this license, visit https://creativecommons.org/licenses/by/4.0/

In cases of disagreement between the two reviewers, discrepancies were resolved through consultation with a third team member (SYL), whose decision was considered final. The overall study selection process is illustrated in the PRISMA flow diagram.⁶ The selection process is illustrated in Fig. 1.

### Scoring

Following completion of the study selection process, all included articles were independently assessed by two reviewers (JSH and SHL) using the 27-item checklist of the PRISMA 2020 statement. The PRISMA checklist served as both the assessment framework and scoring criteria, and its accompanying explanatory document, which provides illustrative examples for several items, was consulted during the evaluation process. Any discrepancies in scoring between the two reviewers were resolved through discussion with a third team member (SYL), whose decision was considered final.

Each item and sub-item was weighted equally; for example, both Item 1 and Item 10a were assigned one point. This approach was adopted because the PRISMA 2020 statement includes additional sub-items compared with the 2009 version, and equal weighting ensured consistent treatment of all checklist components.

An article was awarded a score for each item only if it fully met the criteria specified. However, if the article did not use the exact specific terminology requested but provided a suitable explanation or equivalent alternative, it was still considered to have met the checklist requirements. For example, item 22 requires a “certainty test,” but if the article did not use this specific term but included tests that served the same purpose (e.g. GRADE), or appropriately applied generic measures such as confidence intervals, it was still scored. Additionally, if an article failed to meet the criteria for an item, but provided a reasonable justification for not doing so, depending on the nature of the article, it was still given credit.

Prior to the scoring process, the reviewers underwent structured training and discussion regarding the application of the scoring system. This was undertaken because guideline-based checklists may be subject to individual interpretation, potentially affecting scoring consistency. In addition, several terms and methodological concepts within the PRISMA checklist may not be routinely familiar to junior doctors; these were therefore reviewed and clarified before scoring commenced. Emphasis was placed on adhering as closely as possible to the wording and intent of the PRISMA checklist and its accompanying guidance document to minimise subjective interpretation and ensure consistency across assessments.

### Scoring using AI

The AI platforms used for scoring were OpenAI’s ChatGPT-4.0 and Google’s Gemini Pro 2.5. They are among the most widely used generative AI models and are well suited for autonomous decision-making tasks [16]. Specific instructions were provided to each platform in the form of a “project” for ChatGPT and a “gem” for Gemini. The use of AI took place from May 2025 through the end of August 2025.

The prompt given to the AI was as follows:

1. Evaluate whether the systematic review or meta-analysis is properly written according to the PRISMA checklist.
2. For each point on the checklist, mark “1” if the report meets the criteria and “0” if it does not. Also, score the details for each point.
3. If the exact term required by the checklist is not used, but similar expressions or descriptions are provided, consider that as meeting the requirement.
4. If the criteria are not met, but the report provides a reasonable explanation as to why, assign a score.
5. If the criteria are not strictly met but an alternative approach is used, assign a score.
6. Do not refer to any past conversation history when evaluating the article.

These instructions guided the AI platforms to evaluate the articles in a consistent manner and in accordance with the established PRISMA 2020 checklist. Each AI was able to readily recognise the full text in both PDF and Word documents, as well as the tables and graphs used in the articles.

After giving the scoring instructions to each AI, the scores for each article were recorded. All results were reviewed by the human researcher (SYL) for accuracy. If graphs or tables had not been properly recognised, the plan during manual review was to extract the relevant figures separately and provide them to the AI for recognition; however, testing this approach on a small number of articles showed that such actions did not lead to any significant differences in the results. During initial testing, a small number of articles were evaluated repeatedly; however, before generating the final results, all memory was cleared, and for the study itself, each article was evaluated only once by each AI. Both ChatGPT and Gemini were configured not to retain conversation history before performing the scoring. Each conversation involved up to 10 articles being scored, and if any issues arose during the evaluation process, a new conversation was started. This approach ensured that the evaluation process was carried out efficiently and without the interference of prior conversation history.

### Analysis

All analyses were performed using Microsoft Excel 365. Compliance for each item and the overall compliance of each article were calculated as percentages. Descriptive statistics, such as mean, range, and standard deviation, were also computed. Inter-observer agreement was assessed using Cohen’s Kappa statistic, which was used to evaluate the agreement between the human researchers’ results and the AI results. To assess whether there was any improvement in the results between the 2017 study and the current study, the Mann-Whitney U test was applied.

## Results

A total of 848 articles were identified through the database search, of which 337 duplicates were removed. Following title and abstract screening, a further 230 articles were excluded for not meeting the inclusion criteria. Three additional articles were excluded because the full texts could not be retrieved.

The remaining articles underwent full-text review. During this stage, 45 articles were excluded for not being systematic reviews or meta-analyses, 25 were excluded as non-clinical studies, and 1 was excluded for not being related to ophthalmology. This resulted in a final total of 207 articles included in the study. The study selection process is summarised in Fig. 1. Examples of excluded articles included journal letters, narrative reviews, and expert opinion pieces.

A separate discussion was undertaken regarding the eligibility of 17 borderline articles. During the PRISMA scoring process, there were 563 instances of disagreement between the two reviewers. The inter-rater agreement, calculated using Cohen’s kappa statistic, was 0.68, indicating substantial agreement.

The mean compliance score (%) for the 207 articles was 36.28 (86.37%), with a standard deviation of 6.20 and a range from 11 to 42. A total of 54 articles achieved a full score, and more than 90% of the articles demonstrated over 50% compliance. The scores for each article are detailed in Table 1.

**Table 1:** Compliance Scores (%) of the 207 Articles

*No*	*Title*	*Author (First Author)*	*Journal*	*Published Year*	*Score*	*Percentage*
*1*	*Incidence of sympathetic ophthalmia after intraocular surgery: a systematic review and meta-analysis*	Mohamed S Bondok	*Ophthalmology*	*2024*	*41*	*97.62*
*2*	*International Classification System for Ocular Complications of Anti-VEGF Agents in Clinical Trials.*	Marko M Popovic	*Ophthalmology*	*2024*	*32*	*76.19*
*3*	*Angle-based minimally invasive glaucoma surgery in normal tension glaucoma: A systematic review and meta-analysis.*	Hnin Hnin Oo	*Clin Exp Ophthalmol*	*2024*	*40*	*95.24*
*4*	*Review of emerging trends and projection of future developments in large language models research in ophthalmology.*	Matthew Wong	*Br J Ophthalmol*	*2024*	*39*	*92.86*
*5*	*Safety and Efficacy of Epithelium-Off Corneal Collagen Cross-Linking for the Treatment of Corneal Ectasia: A Report by the American Academy of Ophthalmology.*	Maria S Cortina	*Ophthalmology*	*2024*	*23*	*54.76*
*6*	*Diabetic retinopathy screening through artificial intelligence algorithms: A systematic review.*	Zineb Farahat	*Surv Ophthalmol*	*2024*	*30*	*71.43*
*7*	*Artificial Intelligence and Ophthalmic Clinical Registries.*	Luke Tran	*Am J Ophthalmol*	*2024*	*33*	*78.57*
*8*	*Effect of Intravenous Mannitol on Intraocular Pressure Changes in Vitrectomized and Non-Vitrectomized Eyes: A Systematic Review and Meta-Analysis.*	Hashem Abu Serhan	*Am J Ophthalmol*	*2024*	*39*	*92.86*
*9*	*Effectiveness of Conventional Digital Fundus Photography-Based Teleretinal Screening for Diabetic Retinopathy and Diabetic Macular Edema: A Report by the American Academy of Ophthalmology.*	Christina Y. Weng	*Ophthalmology*	*2024*	*30*	*71.43*
*10*	*The association between vision impairment and incidence of dementia and cognitive impairment: a systematic review and meta-analysis*	Xianwen Shang	*Ophthalmology*	*2024*	*42*	*100.00*
*11*	*A Systematic Review of Ophthalmology Education in Medical Schools: The Global Decline.*	Sascha K R Spencer	*Ophthalmology*	*2024*	*36*	*85.71*
*12*	*Risk factors for complications in resident-performed cataract surgery: A systematic review.*	Chaerim Kang	*Surv Ophthalmol*	*2024*	*37*	*88.10*
*13*	*A systematic review of economic evaluation of artificial intelligence-based screening for eye diseases: From possibility to reality.*	Hongkang Wu	*Surv Ophthalmol*	*2024*	*32*	*76.19*
*14*	*Efficacy and Safety of Lotilaner Ophthalmic Solution 0.25% for the Treatment of Demodex Blepharitis: A Meta-Analysis of Randomized Controlled Trials.*	Syed Muhammad Muneeb Akhtar	*Cont Lens Anterior Eye*	*2024*	*39*	*92.86*
*15*	*The Risk of Sympathetic Ophthalmia Associated with Open-Globe Injury Management Strategies: A Meta-analysis.*	Tim J Patterson	*Ophthalmology*	*2024*	*42*	*100.00*
*16*	*Diagnostic methods for dysthyroid optic neuropathy: A systematic review and analysis.*	Stella Weng Chi Sio	*Surv Ophthalmol*	*2024*	*29*	*69.05*
*17*	*Choroidal thickness in eyes of rheumatoid arthritis patients measured using optical coherence tomography: A systematic review and meta-analysis.*	Sepehr Fekrazad	*Surv Ophthalmol*	*2024*	*41*	*97.62*
*18*	*Diagnostic accuracy of artificial intelligence in detecting retinitis pigmentosa: A systematic review and meta-analysis.*	Ayman Mohammed Musleh	*Surv Ophthalmol*	*2023*	*42*	*100.00*
*19*	*Efficacy and Safety of Lotilaner Ophthalmic Solution (0.25%) for the Treatment of Demodex Blepharitis: A GRADE Assessed Systematic Review and Meta-Analysis of Observational & Experimental Studies.*	Muhammad Talha	*Am J Ophthalmol*	*2024*	*42*	*100.00*
*20*	*Diagnostic Accuracy of Artificial Intelligence-Based Automated Diabetic Retinopathy Screening in Real-World Settings: A Systematic Review and Meta-Analysis.*	Sanil Joseph	*Am J Ophthalmol*	*2024*	*42*	*100.00*
*21*	*The role of intravitreal anti-vascular endothelial growth factor injection in peripheral exudative hemorrhagic chorioretinopathy: A systematic review.*	Akash Gowda	*Surv Ophthalmol*	*2023*	*18*	*42.86*
*22*	*Toric Monofocal Intraocular Lenses for the Correction of Astigmatism during Cataract Surgery: A Report by the American Academy of Ophthalmology.*	Zaina Al-Mohtaseb	*Ophthalmology*	*2023*	*24*	*57.14*
*23*	*Advanced Corneal Imaging in Keratoconus: A Report by the American Academy of Ophthalmology.*	Anthony N Kuo	*Ophthalmology*	*2023*	*29*	*69.05*
*24*	*Two-Year Performance and Safety Results of the MINIject Supraciliary Implant in Patients With Primary Open-Angle Glaucoma: Meta-Analysis of the STAR-I*,* II*,* III Trials.*	*HB Dick*	*Am J Ophthalmol*	*2024*	*35*	*83.33*
*25*	*Inventory of Ocular Pulse Amplitude Values in Healthy Subjects and Patients With Ophthalmologic Illnesses: Systematic Review and Meta-analysis.*	Tania D Shajiei	*Am J Ophthalmol*	*2023*	*42*	*100.00*
*26*	*Secondhand smoke exposure and ocular health: A systematic review.*	Youjuan Zhang	*Surv Ophthalmol*	*2023*	*42*	*100.00*
*27*	*Patient-reported outcomes in patients with vitreous floaters: A systematic literature review.*	Jarinne E Woudstra-de Jong	*Surv Ophthalmol*	*2023*	*26*	*61.90*
*28*	*Efficacy of Adjuvants in Ophthalmic Regional Anesthesia: A Systematic Review and Network Meta-analysis.*	Jan-Philipp Bodenbender	*Am J Ophthalmol*	*2023*	*40*	*95.24*
*29*	*Diagnostic Accuracy of the Amsler Grid Test for Detecting Neovascular Age-Related Macular Degeneration: A Systematic Review and Meta-analysis.*	Jakob Bjerager	*JAMA Ophthalmol*	*2023*	*42*	*100.00*
*30*	*Preoperative evaluations for ophthalmic surgery: A systematic review of 48*,*869 eyes.*	Verina Hanna	*Surv Ophthalmol*	*2023*	*42*	*100.00*
*31*	*The role of near-infrared reflectance imaging in retinal disease: A systematic review.*	Georges Sukkarieh	*Surv Ophthalmol*	*2023*	*17*	*40.48*
*32*	*Corneal Hysteresis for the Diagnosis of Glaucoma and Assessment of Progression Risk: A Report by the American Academy of Ophthalmology.*	Arthur J Sit	*Ophthalmology*	*2023*	*22*	*52.38*
*33*	*Risk of Subsequent Dementia or Alzheimer Disease Among Patients With Age-Related Macular Degeneration: A Systematic Review and Meta-analysis.*	Hou-Ren Tsai	*Am J Ophthalmol*	*2023*	*42*	*100.00*
*34*	*A systematic review on the effects of ROCK inhibitors on proliferation and/or differentiation in human somatic stem cells: A hypothesis that ROCK inhibitors support corneal endothelial healing via acting on the limbal stem cell niche.*	Lloyd R Kopecny	*Ocul Surf*	*2023*	*28*	*66.67*
*35*	*Management of Pain after Photorefractive Keratectomy: A Report by the American Academy of Ophthalmology.*	Walter Allan Steigleman	*Ophthalmology*	*2023*	*28*	*66.67*
*36*	*Standalone XEN45 Gel Stent implantation in the treatment of open-angle glaucoma: A systematic review and meta-analysis.*	Sheng Yang Lim	*Surv Ophthalmol*	*2022*	*39*	*92.86*
*37*	*Antibiotic treatment for dry eye disease related to meibomian gland dysfunction and blepharitis - A review.*	Ragnheidur R Vernhardsdottir	*Ocul Surf*	*2022*	*25*	*59.52*
*38*	*Effectiveness of Laser Refractive Surgery to Address Anisometropic Amblyogenic Refractive Error in Children: A Report by the American Academy of Ophthalmology.*	Kara M Cavuoto	*Ophthalmology*	*2022*	*30*	*71.43*
*39*	*Femtosecond Laser-Assisted Cataract Surgery: A Report by the American Academy of Ophthalmology.*	Charles C Lin	*Ophthalmology*	*2022*	*32*	*76.19*
*40*	*The safety of intracameral phenylephrine - A systematic review.*	Akash Gowda	*Surv Ophthalmol*	*2022*	*11*	*26.19*
*41*	*Sensitivity and specificity of handheld fundus cameras for eye disease: A systematic review and pooled analysis.*	Brittney J Palermo	*Surv Ophthalmol*	*2022*	*31*	*73.81*
*42*	*Corneal Endothelial Cell Density Loss after Glaucoma Surgery Alone or in Combination with Cataract Surgery: A Systematic Review and Meta-analysis.*	Clarissa E H Fang	*Ophthalmology*	*2022*	*42*	*100.00*
*43*	*Virtual reality and augmented reality- emerging screening and diagnostic techniques in ophthalmology: A systematic review.*	Marco King In Ma	*Surv Ophthalmol*	*2022*	*30*	*71.43*
*44*	*Serous retinal detachment as a sign of leukemic choroidopathy: A systematic review.*	Agustina Adaniya	*Surv Ophthalmol*	*2022*	*32*	*76.19*
*45*	*Proptosis and Diplopia Response With Teprotumumab and Placebo vs. the Recommended Treatment Regimen With Intravenous Methylprednisolone in Moderate to Severe Thyroid Eye Disease: A Meta-analysis and Matching-Adjusted Indirect Comparison.*	Raymond S Douglas	*JAMA Ophthalmol*	*2022*	*33*	*78.57*
*46*	*Short-Term efficacy of latanoprostene bunod for the treatment of open-Angle glaucoma and ocular hypertension: A systematic literature review and a network meta-Analysis.*	Paul Harasymowycz	*Br J Ophthalmol*	*2022*	*42*	*100.00*
*47*	*The Incidence of Sympathetic Ophthalmia After Trauma: A Meta-analysis.*	Bonnie He	*Am J Ophthalmol*	*2022*	*42*	*100.00*
*48*	*Ophthalmologic manifestations as the initial presentation of chronic myeloid leukemia: A review.*	Mohamed A Yassin	*Surv Ophthalmol*	*2022*	*17*	*40.48*
*49*	*Meta-analysis of ocular axial length in newborns and infants up to 3 years of age.*	Annabel L W Groot	*Surv Ophthalmol*	*2022*	*33*	*78.57*
*50*	*Adaptive optics ophthalmoscopy: a systematic review of vascular biomarkers.*	Elise Bakke	*Surv Ophthalmol*	*2022*	*34*	*80.95*
*51*	*COVID-19-Related Retinal Micro-vasculopathy - A Review of Current Evidence.*	Kelvin Yc Teo	*Am J Ophthalmol*	*2022*	*34*	*80.95*
*52*	*Characterising the ocular manifestations of COVID-19.*	*Hannah W. Ng*	*Prog Retin Eye Res*	*2024*	*26*	*61.90*
*53*	*Minimally Invasive Glaucoma Surgical Techniques for Open-Angle Glaucoma: An Overview of Cochrane Systematic Reviews and Network Meta-analysis.*	Amanda K Bicket	*JAMA Ophthalmol*	*2021*	*39*	*92.86*
*54*	*Botulinum Toxin Injection for the Treatment of Strabismus: A Report by the American Academy of Ophthalmology.*	Gil Binenbaum	*Ophthalmology*	*2021*	*31*	*73.81*
*55*	*Home- and Office-Based Vergence and Accommodative Therapies for Treatment of Convergence Insufficiency in Children and Young Adults: A Report by the American Academy of Ophthalmology.*	Melinda Y Chang	*Ophthalmology*	*2021*	*31*	*73.81*
*56*	*Multifocal and Accommodating Intraocular Lenses for the Treatment of Presbyopia: A Report by the American Academy of Ophthalmology.*	Julie M Schallhorn	*Ophthalmology*	*2021*	*31*	*73.81*
*57*	*Ophthalmic manifestations of myelin oligodendrocyte glycoprotein-IgG-associated disorder other than optic neuritis: A systematic review.*	Amir R Vosoughi	*Br J Ophthalmol*	*2021*	*31*	*73.81*
*58*	*Deep learning versus ophthalmologists for screening for glaucoma on fundus examination: A systematic review and meta-analysis.*	Mathieu Buisson	*Clin Exp Ophthalmol*	*2021*	*39*	*92.86*
*59*	*Emergence of non-artificial intelligence digital health innovations in ophthalmology: A systematic review.*	Rachel Marjorie Wei Wen Tseng	*Clin Exp Ophthalmol*	*2021*	*40*	*95.24*
*60*	*Office- or Facility-Based Probing for Congenital Nasolacrimal Duct Obstruction: A Report by the American Academy of Ophthalmology.*	David G Morrison	*Ophthalmology*	*2021*	*31*	*73.81*
*61*	*Endoscopic vitreoretinal surgery: Review of current applications and future trends.*	Frank Hiu Ping Lai	*Surv Ophthalmol*	*2021*	*30*	*71.43*
*62*	*Ocular surface disease associated with immune checkpoint inhibitor therapy*	Royce B Park	*Ocul Surf*	*2021*	*30*	*71.43*
*63*	*Intraocular Lens Power Calculation in Eyes with Previous Excimer Laser Surgery for Myopia: A Report by the American Academy of Ophthalmology*	Seth M Pantanelli	*Ophthalmology*	*2021*	*33*	*78.57*
*64*	*Diagnostic Test Accuracy of the Red Reflex Test for Ocular Pathology in Infants: A Meta-analysis*	Yousif Subhi	*JAMA Ophthalmol*	*2021*	*42*	*100.00*
*65*	*Efficacy and Safety of Topical Cysteamine in Corneal Cystinosis: A Systematic Review and Meta-Analysis.*	Sukhmandeep Kaur	*Am J Ophthalmol*	*2021*	*42*	*100.00*
*66*	*The Use of Systemic Steroids in the Treatment of Herpes Zoster Ophthalmicus-Related Ophthalmoplegia: Case Report and Case Meta-analysis.*	Anfei Li	*Am J Ophthalmol*	*2021*	*22*	*52.38*
*67*	*Development of a Core Outcome Set for Clinical Trials in Non-infectious Uveitis of the Posterior Segment.*	Mohammad O Tallouzi	*Ophthalmology*	*2021*	*11*	*26.19*
*68*	*OCT Angiography for the Diagnosis of Glaucoma: A Report by the American Academy of Ophthalmology*	Darrell WuDunn	*Ophthalmology*	*2021*	*17*	*40.48*
*69*	*Ophthalmic involvement of chronic lymphocytic leukemia: A systematic review of 123 cases*	Florence Delestre	*Surv Ophthalmol*	*2021*	*30*	*71.43*
*70*	*Subthreshold laser therapy for macular oedema from branch retinal vein occlusion: Focused review.*	Victor Albert Eng	*Br J Ophthalmol*	*2020*	*29*	*69.05*
*71*	*Imaging Methods for Differentiating Pediatric Papilledema from Pseudopapilledema: A Report by the American Academy of Ophthalmology.*	Melinda Y Chang	*Ophthalmology*	*2020*	*29*	*69.05*
*72*	*Eye involvement in primary central nervous system lymphoma.*	Alexandra L Farrall	*Surv Ophthalmol*	*2020*	*33*	*78.57*
*73*	*Psychosocial impacts of Mendelian eye conditions: A systematic literature review.*	Celeste S D’Amanda	*Surv Ophthalmol*	*2020*	*36*	*85.71*
*74*	*Treatment strategies for Graves’ ophthalmopathy: A network meta-analysis.*	Xiaoxin Zhou	*Br J Ophthalmol*	*2020*	*42*	*100.00*
*75*	*In patients with a positive family history of familial adenomatous polyposis can the condition be diagnosed from the presence of congenital hypertrophy of the retinal pigment epithelium detected via an eye examination: A systematic review.*	Shahzaib Rehan	*Clin Exp Ophthalmol*	*2020*	*35*	*83.33*
*76*	*Accuracy of Autorefraction in Children: A Report by the American Academy of Ophthalmology.*	Lorri B Wilson	*Ophthalmology*	*2020*	*31*	*73.81*
*77*	*Incidence Rate of Secondary Glaucoma Following Congenital Cataract Surgery: An In-Depth Systematic Review and Meta-Analysis.*	Li Li	*Am J Ophthalmol*	*2024*	*41*	*97.62*
*78*	*Intraocular lens power calculation accuracy in patients with keratoconus: Network meta-analysis and systematic review.*	Olga Reitblat	*Clin Exp Ophthalmol*	*2024*	*41*	*97.62*
*79*	*Discordant dry eye disease and chronic pain: A systematic review and meta-analysis.*	M Hoffmann	*Cont Lens Anterior Eye*	*2024*	*40*	*95.24*
*80*	*Temporary Keratoprosthesis and Primary Corneal Graft for Ocular Trauma: A Systematic Review and Meta-Analysis.*	David McMaster	*Am J Ophthalmol*	*2024*	*39*	*92.86*
*81*	*International incidence and temporal trends for rhegmatogenous retinal detachment: A systematic review and meta-analysis.*	Jasmine Yaowei Ge	*Surv Ophthalmol*	*2024*	*38*	*90.48*
*82*	*Effectiveness and Safety of Trabeculectomy Versus Tube Shunt Implantation for Uveitic Glaucoma: A Systematic Review and Meta-Analysis.*	Hashem Abu Serhan	*Am J Ophthalmol*	*2024*	*39*	*92.86*
*83*	*Applications of artificial intelligence in diagnosis of uncommon cystoid macular edema using optical coherence tomography imaging: A systematic review.*	Farhang Hosseini	*Surv Ophthalmol*	*2024*	*34*	*80.95*
*84*	*The efficacy of vitamin D supplementation in dry eye disease: A systematic review and meta-analysis.*	Zeying Chen	*Cont Lens Anterior Eye*	*2024*	*38*	*90.48*
*85*	*Optical coherence tomography angiography measurements in systemic lupus erythematosus: A systematic review and meta-analysis.*	Sepehr Fekrazad	*Surv Ophthalmol*	*2024*	*40*	*95.24*
*86*	*Scoping review of nonsurgical treatment options for macular holes.*	Yong Min Lee	*Surv Ophthalmol*	*2024*	*22*	*52.38*
*87*	*Efficacy of the Subtenon Block in Children Undergoing Strabismus Surgery: A Systematic Review and Meta-Analysis.*	Carolyne Pehora	*Am J Ophthalmol*	*2024*	*42*	*100.00*
*88*	*Factors Affecting Global Adherence for the Uptake of Diabetic Retinopathy Screening: A Systematic Review and Meta-Analysis.*	Masoud Rahmati	*Am J Ophthalmol*	*2024*	*41*	*97.62*
*89*	*Punctal cautery in dry eye disease: A systematic review.*	Ashish Ranjan	*Ocul Surf*	*2024*	*38*	*90.48*
*90*	*Drug Exposure As a Predictor in Diabetic Retinopathy Risk Prediction Models-A Systematic Review and Meta-Analysis.*	Maria Anna Bantounou	*Am J Ophthalmol*	*2024*	*42*	*100.00*
*91*	*A Comprehensive Meta-Analysis on the Role of Analgesics and Anti-Inflammatories in Pan-Retinal Photocoagulation.*	Mateus P Arruda	*Am J Ophthalmol*	*2024*	*42*	*100.00*
*92*	*Light exposure therapy for myopia control: a systematic review and Bayesian network meta-analysis.*	Ebenezer Zaabaar	*Br J Ophthalmol*	*2024*	*39*	*92.86*
*93*	*Influence of serial intravitreal injections on measures of dry eye: A systemic review and meta-analysis.*	Meng Gao	*Cont Lens Anterior Eye*	*2024*	*40*	*95.24*
*94*	*Effectiveness of Propranolol in Preventing Severe Retinopathy of Prematurity: A Comprehensive Systematic Review and Meta-Analysis.*	Muhammad Ashir Shafique	*Am J Ophthalmol*	*2024*	*39*	*92.86*
*95*	*Antibiotics Versus Placebo for Acute Bacterial Conjunctivitis: Findings From a Cochrane Systematic Review.*	Su-Hsun Liu	*Am J Ophthalmol*	*2024*	*42*	*100.00*
*96*	*Measurement of visual function in infantile nystagmus: a systematic review.*	Bader Almagren	*Br J Ophthalmol*	*2024*	*35*	*83.33*
*97*	*Medication-associated orbital inflammation: A systematic review.*	Terence Ang	*Surv Ophthalmol*	*2024*	*27*	*64.29*
*98*	*Fine visuomotor skills in amblyopia: a systematic review and meta-analysis.*	Archayeeta Rakshit	*Br J Ophthalmol*	*2024*	*39*	*92.86*
*99*	*Pars Plana Vitrectomy With Silicone Oil or Gas Tamponade for Uncomplicated Retinal Detachment: A Systematic Review and Meta-Analysis.*	Ryan S Huang	*Am J Ophthalmol*	*2024*	*42*	*100.00*
*100*	*Peripheral Vision in Patients Following Intraocular Lens Implantation: A Systematic Review and Meta-Analysis.*	Pablo Artal	*Am J Ophthalmol*	*2024*	*42*	*100.00*
*101*	*Pediatric and Adolescent Traumatic Macular Hole: A Systematic Review.*	Youssef A H Helmy	*Am J Ophthalmol*	*2024*	*36*	*85.71*
*102*	*The prevention and management of postoperative trachomatous trichiasis: A systematic review.*	Andreas J Kreis	*Surv Ophthalmol*	*2024*	*33*	*78.57*
*103*	*Systematic review of prognostic factors associated with progression to late age-related macular degeneration: Pinnacle study report 2.*	Ahmed M Hagag	*Surv Ophthalmol*	*2024*	*36*	*85.71*
*104*	*The Efficacy and Safety of Standard versus Soft Topical Steroids after Cataract Surgery: A Systematic Review and Meta-analysis.*	Dror Ben Ephraim Noyman	*Ophthalmology*	*2024*	*39*	*92.86*
*105*	*Efficacy of meibomian gland expression combined with Home-Based therapy in the management of dry eye Disease: A systematic review and Meta-Analysis.*	Antonio Ballesteros-Sánchez	*Cont Lens Anterior Eye*	*2024*	*37*	*88.10*
*106*	*Retinal and choroidal changes after anti-VEGF therapy in neovascular-AMD patients: A systematic review and meta-analysis of SD-OCT studies.*	Mohammad Amin Salehi	*Surv Ophthalmol*	*2024*	*42*	*100.00*
*107*	*Evaluating the efficacy and safety of therapeutic interventions for corneal neuropathy: A systematic review.*	Rajni Rajan	*Ocul Surf*	*2024*	*40*	*95.24*
*108*	*Systemic Arterial and Venous Thrombotic Events Associated With Anti-Vascular Endothelial Growth Factor Injections: A Meta-Analysis.*	Aaditeya Jhaveri	*Am J Ophthalmol*	*2024*	*42*	*100.00*
*109*	*Intraocular Lens Power Calculation in Eyes After Myopic Laser Refractive Surgery and Radial Keratotomy: Bayesian Network Meta-analysis.*	Xiaoying Pan	*Am J Ophthalmol*	*2024*	*39*	*92.86*
*110*	*Diagnostic methods for primary vitreoretinal lymphoma: A systematic review.*	Ryan S Huang	*Surv Ophthalmol*	*2024*	*37*	*88.10*
*111*	*NSAIDs and Corticosteroids for the Postoperative Management of Age-Related Cataract Surgery: A Systematic Review and Meta-analysis.*	Joe El Haddad	*Am J Ophthalmol*	*2024*	*39*	*92.86*
*112*	*Exudative Progression of Treatment-Naive Nonexudative Macular Neovascularization in Age-Related Macular Degeneration: A Systematic Review With Meta-Analyses.*	Anne Helene Køllund Nissen	*Am J Ophthalmol*	*2024*	*42*	*100.00*
*113*	*Platelet-rich plasma for treating dry eye disease - A systematic review and meta-analysis.*	Prince Kwaku Akowuah	*Cont Lens Anterior Eye*	*2024*	*42*	*100.00*
*114*	*Antiviral treatment for acute retinal necrosis: A systematic review and meta-analysis.*	Ikhwanuliman Putera	*Surv Ophthalmol*	*2024*	*41*	*97.62*
*115*	*Risk factors for the development or progression of diabetic retinopathy in pregnancy: Meta-analysis and systematic review.*	Swara M Sarvepalli	*Clin Exp Ophthalmol*	*2023*	*42*	*100.00*
*116*	*Postoperative positioning regimens in adults who undergo retinal detachment repair: A systematic review.*	Irina Sverdlichenko	*Surv Ophthalmol*	*2023*	*37*	*88.10*
*117*	*Perfluorohexyloctane in dry eye disease: A systematic review of its efficacy and safety as a novel therapeutic agent.*	Antonio Ballesteros-Sánchez	*Ocul Surf*	*2023*	*34*	*80.95*
*118*	*Comparing interventions for chronic central serous chorioretinopathy: A network meta-analysis.*	Eunice Linh You	*Surv Ophthalmol*	*2023*	*42*	*100.00*
*119*	*Retinal and choroidal changes in AMD: A systematic review and meta-analysis of spectral-domain optical coherence tomography studies.*	Mohammad Amin Salehi	*Surv Ophthalmol*	*2023*	*42*	*100.00*
*120*	*Real-world visual outcomes of cataract surgery based on population-based studies: a systematic review.*	Xiaotong Han	*Br J Ophthalmol*	*2023*	*34*	*80.95*
*121*	*Comparison of Femtosecond Laser Assistance and Manual Trephination in Deep Anterior Lamellar Keratoplasty in the Treatment of Keratoconus: A Meta-Analysis.*	Kaiyue Du	*Am J Ophthalmol*	*2023*	*42*	*100.00*
*122*	*Association between obstructive sleep apnea and floppy eyelid syndrome: A systematic review and metaanalysis.*	Alex Jia Yang Cheong	*Surv Ophthalmol*	*2023*	*40*	*95.24*
*123*	*Prevalence of Diabetic Retinopathy in Indigenous and Non-Indigenous Australians: A Systematic Review and Meta-analysis.*	Mark A Chia	*Ophthalmology*	*2023*	*42*	*100.00*
*124*	*Systematic Review and Meta-analysis: Outcomes After Descemet Membrane Endothelial Keratoplasty Versus Ultrathin Descemet Stripping Automated Endothelial Keratoplasty: Systematic Review and Meta-analysis: DMEK Versus UT-DS(A)EK.*	Anna-Karina B Maier	*Am J Ophthalmol*	*2023*	*39*	*92.86*
*125*	*Efficacy of Ahmed and Baerveldt glaucoma drainage device implantation in the pediatric population: A systematic review and meta-analysis.*	Jeannette Y Stallworth	*Surv Ophthalmol*	*2023*	*42*	*100.00*
*126*	*Extended Depth of Focus Versus Trifocal for Intraocular Lens Implantation: An Updated Systematic Review and Meta-Analysis.*	Mohammad Karam	*Am J Ophthalmol*	*2023*	*42*	*100.00*
*127*	*Anti-tubercular therapy in the treatment of tubercular uveitis: A systematic review and meta-analysis.*	Bjorn Kaijun Betzler	*Surv Ophthalmol*	*2023*	*42*	*100.00*
*128*	*Efficacy and safety of anti-vascular endothelial growth agents for the treatment of polypoidal choroidal vasculopathy: A systematic review and meta-analysis.*	Amin Hatamnejad	*Surv Ophthalmol*	*2023*	*38*	*90.48*
*129*	*Small Incision Lenticule Extraction (SMILE) Versus Laser Assisted Stromal In Situ Keratomileusis (LASIK) for Astigmatism Corrections: A Systematic Review and Meta-analysis.*	Jiaxin Song	*Am J Ophthalmol*	*2023*	*42*	*100.00*
*130*	*Outcomes and Complications of Pars Plana Vitrectomy for Tractional Retinal Detachment in People with Diabetes: A Systematic Review and Meta-analysis.*	Philip McCullough	*JAMA Ophthalmol*	*2023*	*42*	*100.00*
*131*	*Artificial intelligence in screening*,* diagnosis*,* and classification of diabetic macular edema: A systematic review.*	Mohammad Hasan Shahriari	*Surv Ophthalmol*	*2023*	*30*	*71.43*
*132*	*Global Trends in Blindness and Vision Impairment Resulting from Corneal Opacity 1984–2020: A Meta-analysis.*	Erin Y Wang	*Ophthalmology*	*2023*	*33*	*78.57*
*133*	*Placebo Effect and Its Determinants in Ocular Hypotensive Therapy: Meta-analysis and Multiple Meta-regression Analysis.*	Sooyeon Choe	*Ophthalmology*	*2023*	*42*	*100.00*
*134*	*Association of lipid-lowering drugs and antidiabetic drugs with age-related macular degeneration: a meta-analysis in Europeans.*	Matthias M Mauschitz	*Br J Ophthalmol*	*2023*	*35*	*83.33*
*135*	*A Systematic Review and Meta-analysis of Systemic Antihypertensive Medications With Intraocular Pressure and Glaucoma.*	Gareth Leung	*Am J Ophthalmol*	*2023*	*42*	*100.00*
*136*	*GLP-1 receptor agonists and diabetic retinopathy: A meta-analysis of randomized clinical trials.*	Ishani Kapoor	*Surv Ophthalmol*	*2023*	*42*	*100.00*
*137*	*Management of macular oedema due to retinal vein occlusion: An evidence-based systematic review and meta-analysis.*	Elisa E Cornish	*Clin Exp Ophthalmol*	*2023*	*39*	*92.86*
*138*	*Prophylactic intraocular pressure lowering measures in anti-vascular endothelial growth factor therapy: A systematic review and meta-analysis.*	Parnian Arjmand	*Surv Ophthalmol*	*2023*	*42*	*100.00*
*139*	*The Diagnostic Yield of Next Generation Sequencing in Inherited Retinal Diseases: A Systematic Review and Meta-analysis.*	Alexis Ceecee Britten-Jones	*Am J Ophthalmol*	*2023*	*42*	*100.00*
*140*	*The association between keratoconus and allergic eye diseases: A systematic review and meta-analysis.*	Ishith Seth	*Clin Exp Ophthalmol*	*2023*	*41*	*97.62*
*141*	*Pathogenesis of myopic choroidal neovascularization: A systematic review and meta-analysis.*	Xiu Juan Zhang	*Surv Ophthalmol*	*2023*	*41*	*97.62*
*142*	*Efficacy of Repeated Low-Level Red-Light Therapy for Slowing the Progression of Childhood Myopia: A Systematic Review and Meta-analysis.*	Jie Tang	*Am J Ophthalmol*	*2023*	*38*	*90.48*
*143*	*A systematic review and meta-analysis on the efficacy of topical povidone iodine in adenoviral conjunctivitis.*	Julio González Martín-Moro	*Cont Lens Anterior Eye*	*2023*	*39*	*92.86*
*144*	*Stereopsis following surgery in children with congenital and developmental cataracts: A systematic review and meta-analysis.*	Kritika Lohia	*Surv Ophthalmol*	*2023*	*42*	*100.00*
*145*	*Pars plana vitrectomy*,* scleral buckle*,* and pneumatic retinopexy for the management of rhegmatogenous retinal detachment: a meta-analysis.*	Marko M Popovic	*Surv Ophthalmol*	*2022*	*42*	*100.00*
*146*	*Global Estimates of Diabetic Retinopathy Prevalence and Progression in Pregnant Women With Preexisting Diabetes: A Systematic Review and Meta-analysis.*	Felicia Widyaputri	*JAMA Ophthalmol*	*2022*	*39*	*92.86*
*147*	*Identification of presumed corneal neuromas and microneuromas using laser-scanning in vivo confocal microscopy: A systematic review.*	Holly Rose Chinnery	*Br J Ophthalmol*	*2022*	*37*	*88.10*
*148*	*Switching between anti-VEGF agents in the management of refractory diabetic macular edema: A systematic review.*	Kian Madjedi	*Surv Ophthalmol*	*2022*	*27*	*64.29*
*149*	*Global Estimates of Diabetic Retinopathy Prevalence and Progression in Pregnant Individuals with Preexisting Diabetes: A Meta-analysis.*	Felicia Widyaputri	*JAMA Ophthalmol*	*2022*	*40*	*95.24*
*150*	*Performances of Machine Learning in Detecting Glaucoma Using Fundus and Retinal Optical Coherence Tomography Images: A Meta-Analysis.*	Jo-Hsuan Wu	*Am J Ophthalmol*	*2022*	*41*	*97.62*
*151*	*Efficacy and safety of intravitreal and periocular injection of corticosteroids in noninfectious uveitis: a systematic review.*	Rafael José-Vieira	*Surv Ophthalmol*	*2022*	*39*	*92.86*
*152*	*Prevalence and Incidence of Dry Eye and Meibomian Gland Dysfunction in the United States: A Systematic Review and Meta-analysis.*	Paul McCann	*JAMA Ophthalmol*	*2022*	*39*	*92.86*
*153*	*Pars plana vitrectomy versus scleral buckle: A comprehensive meta-analysis of 15*,*947 eyes.*	Arjan S Dhoot	*Surv Ophthalmol*	*2022*	*42*	*100.00*
*154*	*Ocriplasmin for treatment of vitreomacular traction and macular hole: A systematic literature review and individual participant data meta-analysis of randomized*,* controlled*,* double-masked trials.*	Timothy L Jackson	*Surv Ophthalmol*	*2022*	*41*	*97.62*
*155*	*Clinical characteristics and treatment outcomes of cytomegalovirus anterior uveitis and endotheliitis: A systematic review and meta-analysis.*	Rina La Distia Nora	*Surv Ophthalmol*	*2022*	*37*	*88.10*
*156*	*Impact of the Time to Surgery on Visual Outcomes for Rhegmatogenous Retinal Detachment Repair: A Meta-Analysis.*	Amirthan Sothivannan	*Am J Ophthalmol*	*2022*	*42*	*100.00*
*157*	*Efficacy of Thin and Ultrathin Descemet Stripping Automated Endothelial Keratoplasty and Influence of Graft Thickness on Postoperative Outcomes: Systematic Review and Meta-analysis.*	Lauren Béal	*Am J Ophthalmol*	*2022*	*42*	*100.00*
*158*	*Utility of photography for trachoma surveys: A systematic review.*	Fahd Naufal	*Surv Ophthalmol*	*2022*	*35*	*83.33*
*159*	*5-Fluorouracil in primary*,* impending recurrent and recurrent pterygium: Systematic review of the efficacy and safety of a surgical adjuvant and intralesional antimetabolite.*	Brendon W H Lee	*Ocul Surf*	*2022*	*31*	*73.81*
*160*	*Global prevalence and clinical outcomes of tubercular uveitis: a systematic review and meta-analysis.*	Hassan D Alli	*Surv Ophthalmol*	*2022*	*36*	*85.71*
*161*	*Treat-and-extend versus alternate dosing strategies with anti-vascular endothelial growth factor agents to treat center involving diabetic macular edema: A systematic review and meta-analysis of 2*,*346 eyes.*	Gurkaran S Sarohia	*Surv Ophthalmol*	*2022*	*42*	*100.00*
*162*	*Associations of refractive errors and retinal changes measured by optical coherence tomography: A systematic review and meta-analysis.*	Mohammad Amin Salehi	*Surv Ophthalmol*	*2022*	*42*	*100.00*
*163*	*The prevalence of retinopathy in prediabetes: A systematic review.*	Varo Kirthi	*Surv Ophthalmol*	*2022*	*42*	*100.00*
*164*	*Retinal displacement following rhegmatogenous retinal detachment: A systematic review and meta-analysis.*	Ryan H Mason	*Surv Ophthalmol*	*2022*	*40*	*95.24*
*165*	*Prevalence of diabetic macular edema based on optical coherence tomography in people with diabetes: A systematic review and meta-analysis.*	James H B Im	*Surv Ophthalmol*	*2022*	*36*	*85.71*
*166*	*Impact of aging on the pathophysiology of dry eye disease: A systematic review and meta-analysis.*	Koji Kitazawa	*Ocul Surf*	*2022*	*31*	*73.81*
*167*	*Association Between Visual Acuity and Residual Retinal Fluid Following Intravitreal Anti-Vascular Endothelial Growth Factor Treatment for Neovascular Age-Related Macular Degeneration: A Systematic Review and Meta-analysis.*	Nikhil S Patil	*JAMA Ophthalmol*	*2022*	*40*	*95.24*
*168*	*OCT-Angiography detects longitudinal microvascular changes in glaucoma: A systematic review.*	Ana Miguel	*Br J Ophthalmol*	*2022*	*36*	*85.71*
*169*	*Alcohol*,* Intraocular Pressure*,* and Open-Angle Glaucoma: A Systematic Review and Meta-analysis.*	Kelsey V Stuart	*Ophthalmology*	*2022*	*42*	*100.00*
*170*	*Degree of Myopia and Glaucoma Risk: A Dose-Response Meta-analysis.*	Ahnul Ha	*Am J Ophthalmol*	*2022*	*41*	*97.62*
*171*	*Corneal collagen crosslinking in keratoconus: Epithelium on or off? A systematic review and meta-analysis.*	Grace A Borchert	*Clin Exp Ophthalmol*	*2024*	*39*	*92.86*
*172*	*Conclusions from a systematic review of artificial intelligence deep learning algorithms for diagnosing retinopathy of prematurity: recommendations for future artificial intelligence algorithms.*	*Bai*,* Dai*,* Carty*	*Clin Exp Ophthalmol*	*2022*	*33*	*78.57*
*173*	*Benefits and risks of orthokeratology treatment: A systematic review and meta-analysis.*	Lauren Sartor	*Ophthalmology*	*2024*	*42*	*100.00*
*174*	*Structural changes associated to orthokeratology: A systematic review.*	Alicia Sánchez-García	*Cont Lens Anterior Eye*	*2021*	*33*	*78.57*
*175*	*Accuracy of optical coherence tomography for diagnosing glaucoma: An overview of systematic reviews.*	Manuele Michelessi	*Br J Ophthalmol*	*2021*	*39*	*92.86*
*176*	*Ocular Injury Associated With Prone Positioning in Adult Critical Care: A Systematic Review and Meta-Analysis.*	Timothy J Patterson	*Am J Ophthalmol*	*2021*	*39*	*92.86*
*177*	*Long-term natural history of visual acuity in eyes with choroideremia: A systematic review and meta-analysis of data from 1004 individual eyes.*	Liangbo L Shen	*Br J Ophthalmol*	*2021*	*30*	*71.43*
*178*	*Efficacy of intracameral antibiotics following manual small incision cataract surgery in reducing the rates of endophthalmitis: A meta-analysis.*	Khizar Rana	*Clin Exp Ophthalmol*	*2021*	*35*	*83.33*
*179*	*Transepithelial corneal collagen cross-linking using iontophoresis versus the Dresden protocol in progressive keratoconus: A meta-analysis.*	Kelvin H Wan	*Clin Exp Ophthalmol*	*2021*	*35*	*83.33*
*180*	*Early Detection of Microvascular Impairments With Optical Coherence Tomography Angiography in Diabetic Patients Without Clinical Retinopathy: A Meta-analysis.*	Bilei Zhang	*Am J Ophthalmol*	*2021*	*37*	*88.10*
*181*	*Effects of Internal Limiting Membrane Peel for Idiopathic Epiretinal Membrane Surgery: A Systematic Review of Randomized Controlled Trials.*	Parsa Mehraban Far	*Am J Ophthalmol*	*2021*	*41*	*97.62*
*182*	*Microinvasive glaucoma surgery: A review of 3476 eyes.*	Prem Nichani	*Surv Ophthalmol*	*2021*	*35*	*83.33*
*183*	*Interventions for Demodex blepharitis and their effectiveness: A systematic review and meta-analysis.*	Dayron F Martínez-Pulgarín	*Cont Lens Anterior Eye*	*2021*	*34*	*80.95*
*184*	*Genetic associations of central serous chorioretinopathy: a systematic review and meta-analysis.*	Zhen Ji Chen	*Br J Ophthalmol*	*2022*	*36*	*85.71*
*185*	*Intravitreal antivascular endothelial growth factor injection versus laser photocoagulation for retinopathy of prematurity: A meta-analysis of 3*,*701 eyes.*	Marko M Popovic	*Surv Ophthalmol*	*2021*	*41*	*97.62*
*186*	*Safety of Receiving Anti-Vascular Endothelial Growth Factor Intravitreal Injection in Office-Based vs. Operating Room Settings: A Meta-analysis.*	Tong Li	*JAMA Ophthalmol*	*2021*	*37*	*88.10*
*187*	*Transepithelial versus Epithelium-off Corneal Collagen Cross-linking for Corneal Ectasia: A Systematic Review and Meta-analysis.*	Siddharth Nath	*Ophthalmology*	*2021*	*42*	*100.00*
*188*	*Cardiovascular Adverse Events with Intravitreal Anti-Vascular Endothelial Growth Factor Drugs: A Systematic Review and Meta-analysis of Randomized Clinical Trials.*	Nadège Ngo Ntjam	*JAMA Ophthalmol*	*2021*	*39*	*92.86*
*189*	*Diagnostic Test Accuracy of the Red Reflex Test for Ocular Pathology in Infants: A Meta-analysis.*	Yousif Subhi	*JAMA Ophthalmol*	*2021*	*42*	*100.00*
*190*	*The Global Extent of Undetected Glaucoma in Adults: A Systematic Review and Meta-analysis.*	Zhi Soh	*Ophthalmology*	*2021*	*40*	*95.24*
*191*	*Neurodevelopmental Outcomes after Bevacizumab Treatment for Retinopathy of Prematurity: A Meta-analysis.*	Chia-Ying Tsai	*Ophthalmology*	*2021*	*42*	*100.00*
*192*	*Serum vitamin D and age-related macular degeneration: Systematic review and meta-analysis.*	André Ferreira	*Surv Ophthalmol*	*2021*	*35*	*83.33*
*193*	*Laser Trabeculoplasty for Open-Angle Glaucoma: A Systematic Review and Network Meta-Analysis.*	Rouxi Zhou	*Am J Ophthalmol*	*2021*	*37*	*88.10*
*194*	*Choroidal Thickness in Diabetic Patients Without Diabetic Retinopathy: A Meta-analysis.*	Hiroaki Endo	*Am J Ophthalmol*	*2020*	*37*	*88.10*
*195*	*Association between vitamin D and dry eye disease: A systematic review and meta-analysis of observational studies.*	Gholamreza Askari	*Cont Lens Anterior Eye*	*2020*	*40*	*95.24*
*196*	*Outcomes of Limbal Stem Cell Transplant: A Meta-analysis.*	Qihua Le	*JAMA Ophthalmol*	*2020*	*35*	*83.33*
*197*	*Autologous limbal stem cell transplantation: A systematic review of clinical outcomes with different surgical techniques.*	Swapna S Shanbhag	*Br J Ophthalmol*	*2020*	*37*	*88.10*
*198*	*Systematic review of potential causes of intraocular lens opacification.*	Joaquín Fernández	*Clin Exp Ophthalmol*	*2020*	*35*	*83.33*
*199*	*Sclerotherapy for low-flow vascular malformations of the orbital and periocular regions: Systematic review and meta-analysis.*	Lucio De Maria	*Surv Ophthalmol*	*2020*	*35*	*83.33*
*200*	*Central corneal basal cell density and nerve parameters in ocular surface disease and limbal stem cell deficiency: A review and meta-analysis.*	Pradipta Bhattacharya	*Br J Ophthalmol*	*2020*	*34*	*80.95*
*201*	*Risk of Death Associated with Intravitreal Anti-Vascular Endothelial Growth Factor Therapy: A Systematic Review and Meta-analysis.*	Michele Reibaldi	*JAMA Ophthalmol*	*2020*	*32*	*76.19*
*202*	*Multifocal spectacles in childhood myopia: Are treatment effects maintained? A systematic review and meta-analysis.*	Dinesh Kaphle	*Surv Ophthalmol*	*2020*	*35*	*83.33*
*203*	*Adherence of patients with diabetic macular oedema to intravitreal injections: A systematic review.*	Monique A Rose	*Clin Exp Ophthalmol*	*2020*	*31*	*73.81*
*204*	*Prevalence of myopic macular degeneration worldwide: A systematic review and meta-analysis.*	Minjie Zou	*Br J Ophthalmol*	*2020*	*34*	*80.95*
*205*	*Biologic therapy for Behcet’s uveitis: A systematic review.*	Perpetual Uke	*Br J Ophthalmol*	*2020*	*31*	*73.81*
*206*	*Diagnostic criteria for limbal stem cell deficiency before surgical intervention-A systematic literature review and analysis.*	Qihua Le	*Surv Ophthalmol*	*2020*	*29*	*69.05*
*207*	*Detection of choroidal vascular features in diabetic patients without clinically visible diabetic retinopathy by optical coherence tomography angiography: A systemic review and meta-analysis. [Review]*	*Q Zhao*	*Surv Ophthalmol*	*2024*	*36*	*85.71*

**Fig. 2:**
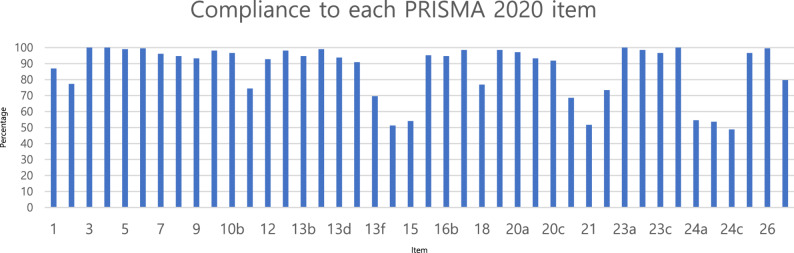
Compliance Rates (%) by PRISMA Item and Subpoint

Out of the 27 items and 42 subpoints, 6 items (3, 4, 6, 23a, 23d, and 26) showed 100% compliance, while 29 items demonstrated over 90% compliance. On the other hand, relatively lower scores were observed in items 13f, 14, 15, 20d, 21, 24a, 24b, and 24c where compliance rates were observed to be below 70%. Detailed compliance results for each item are summarized in Table 2 and Fig. 2.

**Table 2:** Compliance Rates for Each Reporting Item and Subpoint

Item number	Checklist item	Point	Percentage
1	Identify the report as a systematic review.	180	87
2	See the PRISMA 2020 for Abstracts checklist.	160	77
3	Describe the rationale for the review in the context of existing knowledge.	207	100
4	Provide an explicit statement of the objective(s) or question(s) the review addresses.	207	100
5	Specify the inclusion and exclusion criteria for the review and how studies were grouped for the syntheses.	205	99
6	Specify all databases, registers, websites, organisations, reference lists and other sources searched or consulted to identify studies. Specify the date when each source was last searched or consulted.	206	100
7	Present the full search strategies for all databases, registers and websites, including any filters and limits used.	199	96
8	Specify the methods used to decide whether a study met the inclusion criteria of the review, including how many reviewers screened each record and each report retrieved, whether they worked independently, and if applicable, details of automation tools used in the process.	196	95
9	Specify the methods used to collect data from reports, including how many reviewers collected data from each report, whether they worked independently, any processes for obtaining or confirming data from study investigators, and if applicable, details of automation tools used in the process.	193	93
10a	List and define all outcomes for which data were sought. Specify whether all results that were compatible with each outcome domain in each study were sought (e.g. for all measures, time points, analyses), and if not, the methods used to decide which results to collect.	203	98
10b	List and define all other variables for which data were sought (e.g. participant and intervention characteristics, funding sources). Describe any assumptions made about any missing or unclear information.	200	97
11	Specify the methods used to assess risk of bias in the included studies, including details of the tool(s) used, how many reviewers assessed each study and whether they worked independently, and if applicable, details of automation tools used in the process.	154	74
12	Specify for each outcome the effect measure(s) (e.g. risk ratio, mean difference) used in the synthesis or presentation of results.	192	93
13a	Describe the processes used to decide which studies were eligible for each synthesis (e.g. tabulating the study intervention characteristics and comparing against the planned groups for each synthesis (item #5)).	203	98
13b	Describe any methods required to prepare the data for presentation or synthesis, such as handling of missing summary statistics, or data conversions.	196	95
13c	Describe any methods used to tabulate or visually display results of individual studies and syntheses.	205	99
13d	Describe any methods used to synthesize results and provide a rationale for the choice(s). If meta-analysis was performed, describe the model(s), method(s) to identify the presence and extent of statistical heterogeneity, and software package(s) used.	194	94
13e	Describe any methods used to explore possible causes of heterogeneity among study results (e.g. subgroup analysis, meta-regression).	188	91
13f	Describe any sensitivity analyses conducted to assess robustness of the synthesized results.	144	70
14	Describe any methods used to assess risk of bias due to missing results in a synthesis (arising from reporting biases).	106	51
15	Describe any methods used to assess certainty (or confidence) in the body of evidence for an outcome.	112	54
16a	Describe the results of the search and selection process, from the number of records identified in the search to the number of studies included in the review, ideally using a flow diagram.	197	95
16b	Cite studies that might appear to meet the inclusion criteria, but which were excluded, and explain why they were excluded.	196	95
17	Cite each included study and present its characteristics.	204	99
18	Present assessments of risk of bias for each included study.	159	77
19	For all outcomes, present, for each study: (a) summary statistics for each group (where appropriate) and (b) an effect estimate and its precision (e.g. confidence/credible interval), ideally using structured tables or plots.	204	99
20a	For each synthesis, briefly summarise the characteristics and risk of bias among contributing studies.	201	97
20b	Present results of all statistical syntheses conducted. If meta-analysis was done, present for each the summary estimate and its precision (e.g. confidence/credible interval) and measures of statistical heterogeneity. If comparing groups, describe the direction of the effect.	193	93
20c	Present results of all investigations of possible causes of heterogeneity among study results.	190	92
20d	Present results of all sensitivity analyses conducted to assess the robustness of the synthesized results.	142	69
21	Present assessments of risk of bias due to missing results (arising from reporting biases) for each synthesis assessed.	107	52
22	Present assessments of certainty (or confidence) in the body of evidence for each outcome assessed.	152	73
23a	Provide a general interpretation of the results in the context of other evidence.	207	100
23b	Discuss any limitations of the evidence included in the review.	204	99
23c	Discuss any limitations of the review processes used.	200	97
23d	Discuss implications of the results for practice, policy, and future research.	207	100
24a	Provide registration information for the review, including register name and registration number, or state that the review was not registered.	113	55
24b	Indicate where the review protocol can be accessed, or state that a protocol was not prepared.	111	54
24c	Describe and explain any amendments to information provided at registration or in the protocol.	101	49
25	Describe sources of financial or non-financial support for the review, and the role of the funders or sponsors in the review.	200	97
26	Declare any competing interests of review authors.	206	100
27	Report which of the following are publicly available and where they can be found: template data collection forms; data extracted from included studies; data used for all analyses; analytic code; any other materials used in the review.	165	80

**Fig. 3:**
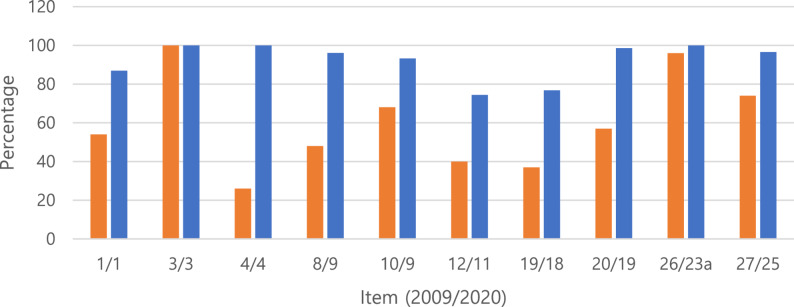
Comparison of Compliance Rates Between the 2017 Study and the Present Study for Shared PRISMA Items

Additionally, the results were compared with the 2017 study. The Mann-Whitney U test yielded a score of 3290, a z-score of 9.59, a p-value of < 0.00001 at (*p* < 0.05), effect size 0.550 (i.e. large), and 95% CI 0.466, 0.624. This indicates that the results of the current study show a statistically significant improvement in compliance to the PRISMA checklist in comparison to the 2017 study. Unfortunately, the 2017 study used the 2009 version of the PRISMA statement, making a direct comparison impossible. However, several items remained largely unchanged and were also included in the 2020 version. Therefore, those items were compared separately, and the results are presented in Table 3 and Fig. 3. Although this represents a more simplified comparison, it can be seen that, across all items, the results of the present study are either comparable to or improved compared with those of the 2017 study.

**Table 3:**
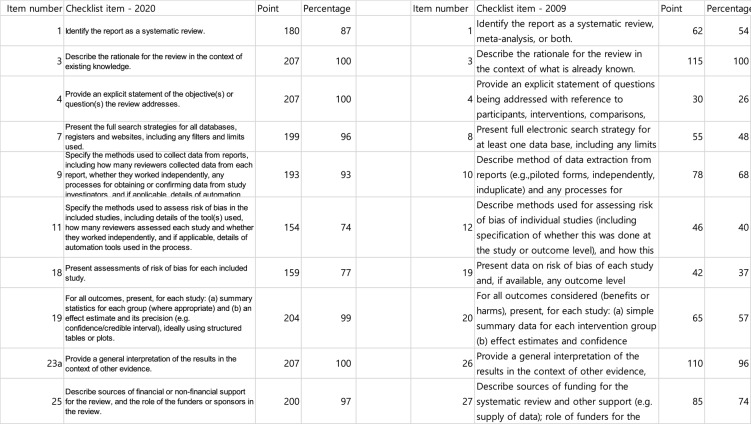
Comparison of Compliance Rates for PRISMA Items Shared Between the 2017 Study and the Present Study

In terms of AI-based scoring, the mean compliance for the 207 articles was 34.3 (81.68%) with standard deviation of 6.11 when using ChatGPT, and 33.1 (78.72%) with standard deviation of 6.56 when using Gemini. For ChatGPT, the highest-scoring items were 3, 4, 23d, and 26, all showing 100% compliance, while the lowest-scoring items were item 15 (26%) and item 24c (29%). For Gemini, the highest-scoring items included item 3 (100%), item 2 (99%), 23a (99%), and 23d (99%), while the lowest-scoring items were item 15 (28%) and item 22 (33%).

Regarding the agreement between AI and human scoring, the Cohen’s Kappa statistic was 0.63 (moderate agreement) when using ChatGPT, and 0.537 (moderate agreement) when using Gemini.

## Discussion

The results suggest a significant improvement in compliance with the PRISMA statement compared to the previous study. Although the sample size in the current study is larger than that of the 2017 study, the level of significance observed suggests that there has been a substantial change in ophthalmology reporting quality over the past few years. Reporting quality in systematic reviews and meta-analyses across other various medical fields, including emergency medicine, cardiology, and rehabilitation, over the past few years has also improved [17–19]. Other studies also demonstrate that the introduction of PRISMA 2020 has led to improvements in the reporting quality of systematic reviews and meta-analyses [9, 20].

Overall, this study demonstrates a high level of compliance, in particular, the areas related to the article’s background and rationale (items 3 and 4), as well as in the description of the process for selecting articles to be included in the systematic review or meta-analysis, the criteria for article selection, and the data extraction process (items 5 to 10b, and items 16a, 17), along with the reporting of the article’s limitations (items 23a to 23c). These areas potentially represent a more detailed and thorough understanding in general regarding the process of systematic reviews and meta-analysis, which in turn, has led to more accurate and comprehensive reporting. While these items were highlighted as areas of strength in the 2017 study, the results here, that not only was this strength retained but in-fact enhanced.

An interesting observation is found in the area of describing the objectives (item 4), where, in the 2017 study, a relatively low score (compliance 30, 26%) was recorded, where as in this study, 100% compliance was achieved. This is primarily due to changes in the description of the item. In the 2009 version of the PRISMA statement, the item was specifically limited to the “Description of PICOS (Participants, Interventions, Comparisons, and Study design),” while the 2020 version allowed for a broader interpretation with the instruction to “Provide an explicit statement of the objective(s) or question(s) the review addresses.” This change in description allowed for greater flexibility in how the objectives could be reported. However, given that the 2020 version emphasized an “explicit statement,” only those articles that provided a clear and appropriate description were awarded points. It is therefore likely that the improvement in reporting quality contributed to the 100% compliance observed.

On the other hand, areas that showed relatively lower scores included the description of the risk of bias (item 11) and the assessment of bias (item 21) due to missing data or results. Compliance with items related to bias was also noted to have been comparatively poor in the 2017 study. This is not an issue limited to articles published in ophthalmology; studies, including those from the Cochrane Library, have pointed out that non-reporting bias and selective reporting bias are insufficiently reported, especially in their impact on meta-analyses [21–24]. Non-reporting bias and selective reporting bias are known to hinder the accurate assessment of treatment efficacy (benefit) or harm. For example, harmful outcomes or side effects are often underreported, leading to an overestimation of the true safety of a treatment [21, 22]. Furthermore, selective reporting can result in discrepancies between original studies or analysis plans (protocols) and the results actually reported, which ultimately prevents proper understanding of the true effects and side effects of a study, diminishing reproducibility [25, 26]. Selective reporting bias and non-reporting bias are challenging to detect and correct (e.g. sensitivity analysis, funnel plots), making them fundamental risks that greatly undermine the transparency of clinical research and evidence synthesis [22, 27]. Therefore, the Cochrane Handbook and the latest PRISMA guidelines explicitly state that any selective or non-reporting bias should always be classified as “high risk,” and caution should be exercised when applying meta-analyses in such cases [6, 21].

Another area with relatively low compliance was sensitivity analysis (items 13f, 20c), which was introduced as a new item in the PRISMA 2020 statement. This issue is not limited to ophthalmology but is common across various fields [28, 29]. The challenge lies in that, depending on the research field or the nature of the systematic review/meta-analysis, including analysis on sensitivity may not always be feasible [28]. Researchers have different criteria’s for determining which variables (e.g., quality, bias, missing outcomes, analytical methods) to include in a sensitivity analysis, and there is often inconsistency in thresholds or inclusion/exclusion criteria, making reporting difficult [6, 28–30].

Nonetheless, sensitivity analysis plays a crucial role in improving clinical interpretation, practical application, and the reliability of decision-making by verifying whether the main conclusions remain consistent when various assumptions or methodological choices—such as analytical methods, inclusion/exclusion criteria, or data handling methods—are altered [31, 32]. Both PRISMA 2020 and the Cochrane Handbook emphasize the reporting of sensitivity analysis in their guidelines [6, 21]. However, many systematic reviews do not explicitly report sensitivity analyses related to data shortages (e.g., studies with missing results or unmeasured outcomes), and due to a lack of consistent application of reporting guidelines and insufficient training, researchers may not recognise it as a necessary component [29]. In clinical papers especially, there often tends to be favouritism toward results-focused reporting, which often leads to a reduction in the explanation of methodological robustness, such as sensitivity analysis [31]. When the results of a sensitivity analysis diverge from the primary analysis, leading to more complex interpretations, the tendency to either gloss over these differences or completely omit its reporting can lead to insufficient reporting practices, which remains a significant issue [33].

The final area with comparatively low compliance was related to research registration (items 24a to 24c), which in the 2009 version of the PRISMA statement was a single item (item 5) but was expanded to a major category with three subpoints in the updated 2020 checklist. In the 2017 study, this category also showed a low score (10, 9%). Previously, we attributed this to a lack of awareness and promotion of platforms where research could be registered, as well as journals not explicitly requiring it. However, several years later, it is no longer justifiable to blame the lack of platform promotion alone. In fact, 7 of the 11 journals included in this study now either mandate or encourage registration (Table 4). As a result, the absolute compliance rate has increased compared to the past, but it still remains an area that requires further attention.

**Table 4:** Research registration requirements by journal (as of 2024)

Journal Name	Requirement for Clinical Trial / Research Registration	Notes / Policy Summary
Progress in Retinal and Eye Research	Not required (may apply if clinical trial)	Primarily reviews/basic research; no explicit registration policy.
Ophthalmology	Mandatory for clinical trials / interventional studies	Follows ICMJE guidelines; must register (e.g., ClinicalTrials.gov).
JAMA Ophthalmology	Mandatory for clinical trials / interventional studies	Trial registry, ID, and URL must be stated in abstract/methods.
Ocular Surface	Not required / registration and ethics approval required for clinical trials (explicit rule unclear)	Generally requires trial registration for original interventional studies.
Survey of Ophthalmology	Not required	Mostly reviews; author guidelines do not mandate registration.
Annual Review of Vision Science	Not required	Publishes review articles exclusively; no related policy.
Clinical and Experimental Ophthalmology	Recommended / Ethics approval required	Endorses ICMJE recommendations but lacks explicit mandatory rule.
Contact Lens and Anterior Eye	Not required	Focus on ethical standards and originality; registration encouraged.
American Journal of Ophthalmology	Recommended / (almost mandatory) for clinical trials	To avoid duplication and non-disclosure; registration preferred.
British Journal of Ophthalmology	Mandatory for clinical trials	Follows BMJ Group’s trial registration policy; strict requirements.
Asia Pacific Journal of Ophthalmology	Mandatory for clinical trials	Requires registration of RCTs and prospective trials.

1. Elsevier. *Guide for Authors – Progress in Retinal and Eye Research*. Amsterdam: Elsevier; 2024 [cited 2025 Aug 10]. Available from: https://www.elsevier.com/journals/progress-in-retinal-and-eye-research

2. American Academy of Ophthalmology. *Instructions for Authors – Ophthalmology*. San Francisco: American Academy of Ophthalmology; 2024 [cited 2025 Aug 10]. Available from: https://www.aaojournal.org/content/authorinfo

3. JAMA Network. *Instructions for Authors – JAMA Ophthalmology*. Chicago: American Medical Association; 2024 [cited 2025 Aug 10]. Available from: https://jamanetwork.com/journals/jamaophthalmology/pages/instructions-for-authors

4. Elsevier. *Guide for Authors – The Ocular Surface*. Amsterdam: Elsevier; 2024 [cited 2025 Aug 10]. Available from: https://www.journals.elsevier.com/the-ocular-surface

5. Elsevier. *Guide for Authors – Survey of Ophthalmology*. Amsterdam: Elsevier; 2024 [cited 2025 Aug 10]. Available from: https://www.journals.elsevier.com/survey-of-ophthalmology

6. Annual Reviews. *Instructions for Authors – Annual Review of Vision Science*. Palo Alto: Annual Reviews; 2024 [cited 2025 Aug 10]. Available from: https://www.annualreviews.org/journal/vision

7. Wiley. *Author Guidelines – Clinical and Experimental Ophthalmology*. Hoboken: Wiley; 2024 [cited 2025 Aug 10]. Available from: https://onlinelibrary.wiley.com/journal/14429071

8. Elsevier. *Guide for Authors – Contact Lens and Anterior Eye*. Amsterdam: Elsevier; 2024 [cited 2025 Aug 10]. Available from: https://www.journals.elsevier.com/contact-lens-and-anterior-eye

9. Elsevier. *Guide for Authors – American Journal of Ophthalmology*. Amsterdam: Elsevier; 2024 [cited 2025 Aug 10]. Available from: https://www.ajo.com/content/authorinfo

10. BMJ Publishing Group. *Instructions for Authors – British Journal of Ophthalmology*. London: BMJ; 2024 [cited 2025 Aug 10]. Available from: https://bjo.bmj.com/pages/authors

11. Springer. *Guide for Authors – Asia Pacific Journal of Ophthalmology*. Singapore: Springer Nature; 2024 [cited 2025 Aug 10]. Available from: [journal homepage]

One potential reason for this low compliance is that the importance of research registration is not recognised. The role of registration goes beyond enhancing the transparency of research; it helps establish global standards in the relevant field and in medicine as a whole, reducing unnecessary duplication, and fosters collaboration by connecting researchers conducting similar studies [29, 34, 35]. This is further reflected in how research-related training in medical schools often does not emphasise registration [36]. Therefore, this issue requires efforts from journals, institutions, and policymakers – all of whom are making active efforts to do so by promoting the importance of registration. It is hoped that over time, the rate of registration will continue to rise [37]. In the immediate term however, registration may not significantly impact the tasks that researchers are focusing on and thus may be overlooked.

Another important point of this study was the use of AI – and its potential role in assessment of quality in modern research articles. This study demonstrated that the AI scoring of the articles showed a reasonable level of agreement against its human counter parts. The key advantage of using AI for evaluation, compared to manual scoring, is its overwhelming speed. AI has the ability to analyse a single study in mere seconds to minutes, in contrast to the relatively slow process of a human assessor – which depending on the varying degree of experience can take between several hours [38, 39]. This stark contrast in speed illustrates the potential efficiency benefits AI offers in large-scale evaluations.

However, this study also highlighted current limitations of AI use in research. In this study, the process required analysing a large number of articles multiple times using the same method, which following repeated cycles, there were instances where AI would duplicate previous answers or, in some cases, fail to provide a response altogether. This issue is not limited to medical-related queries but appears to be a common occurrence for many general users of AI or Large Language Model (LLMs) across various fields [40, 41] It highlights a limitation in the AI’s ability to maintain consistency and accuracy when processing repetitive tasks, which highlights a potential issue in the use of AI especially in large-scale analyses or when ensuring reliability in responses.

Additionally, due to the nature of this study, the two LLM or AI platforms were required to “read” a large number of PDF or DOC files, but it was noted during the study that there were instances where it struggled to accurately interpret graphs or more visual representation of results. For example, in item 22, “Present assessments of certainty (or confidence) in the body of evidence for each outcome assessed,” where the results involving certainty were frequently displayed in graphs, both ChatGPT and Gemini repeatedly failed to recognise the results. In fact, the compliance score for item 22, as assessed by humans, was 152 (73%), while ChatGPT scored only 66 (32%), and Gemini scored 56 (27%). This issue is recognised as one of the broader limitations of AI systems, particularly when dealing with complex visual data such as graphs or figures, which remain a challenge for AI-based tools to interpret accurately [42].

The most significant issue encountered in this study was the repeated occurrence of different results when the same paper was analysed with the same question multiple times. Although this did not have a major impact on the overall score, there were inconsistencies in certain details, indicating that relying solely on AI to understand an article could be problematic. AI systems, including ChatGPT and Gemini, may produce different outcomes for the same question depending on the user’s past interactions [43]. However, despite instructing the AI in this study to not reference previous records, varying results continued to occur, which suggests that full reliance on AI judgment remains at present, impractical. While this field is one of rapid advancements, and the reliability of AI or LLMs will over time dramatically improve, it is essential to acknowledge that ultimate responsibility of thoroughly understanding, coordinating, and making judgments on the research lies with the researcher. The researcher’s expertise and critical thinking must not be overlooked, even as AI tools become more integrated into the research process [44, 45].

The increasing reliance on AI in medical research, not just in this study but across all fields, is a growing trend. Given the rapid pace of AI development, this will likely intensify [46]. As a result, the way AI is utilised in research will become a continued important aspect of future studies. It is anticipated that future updates to the PRISMA guidelines should include specific items related to AI usage, addressing its role and the potential challenges in ensuring transparency, consistency, and accuracy in research findings. This inclusion will help standardise AI applications and improve the overall quality of reporting in systematic reviews and meta-analyses.

## Limitations

This study has several limitations. First, the evaluation of systematic reviews and meta-analyses was based solely on the PRISMA statement. While this focus on reporting quality was intentional for the scope of this study, it is worth noting that using additional tools, such as AMSTAR 2, to assess the methodological quality of the systematic reviews and meta-analyses could potentially have broadened the evaluation spectrum [47]. This may have provided a greater, comprehensive insight and potentially led to additional discussion points.

Additionally, similar to the previous study, only articles published in 11 journals based on the greatest impact factor were included in this study. The intention behind using this method was to minimise bias when comparing the current study with past research by maintaining consistency in methodology. However, this approach also meant that studies published in journals with lower impact factors were excluded from the study in its entirety, which may limit the generalisability of the findings and potentially introduce a selection bias.

In contrast to the previous study, where proportional scores were assigned to subpoints, this study assigned the same weight to subpoints as to the main items. This change was an unavoidable adjustment due to the increased number of subpoints in the PRISMA 2020 statement compared to the 2009 version. However, it is also true that this alteration could introduce structural bias when comparing the results with those of the past study, as the scoring method differs between the two analyses.

In addition, articles were awarded credit even when they did not use the specific terminology employed in the PRISMA checklist, provided that they performed an equivalent procedure or clearly explained why such a procedure was not necessary. This approach may introduce a confounding error and potentially inflate the compliance rate. However, because the primary objective of this study was to assess reporting quality rather than to identify the use of specific analytical methods, greater weight was placed on the authors’ demonstrated understanding and explanation of each relevant item within the article. To further mitigate this potential source of error, each article was independently assessed by two reviewers, and discrepancies were subsequently reviewed and resolved by a third assessor.

Finally, differences in the understanding or interpretation of each item by the scorers were evident, which, while not altering the essence of the evaluated articles, led to variations in the scores. To minimise this, a team member who was not involved in the initial scoring was tasked with evaluating the final results and in cases where score discrepancies were significant, discussions were held to come to an ultimate decision on the scores. However, it is important to note that such differences in interpretation can also witnessed among the authors of the articles themselves. For example, the degree of an “explicit statement,” as seen in item 4, can be interpreted differently by individuals. One author may feel that a relatively simple description meets the requirement, while another author may believe that a more detailed explanation is necessary.

## Conclusion

This study ultimately reveals significant improvements in the reporting quality of systematic reviews and meta-analyses in ophthalmology, particularly with the adoption of the PRISMA 2020 checklist. Compared to the 2017 study, the results show a substantial increase in compliance, especially in areas related to the background and rationale, selection criteria, limitations, and the clarity of objectives. Despite these improvements, challenges persist in areas such as bias reporting, sensitivity analysis, and research registration, where compliance remains relatively low. Most notably, bias reporting continues to be a weak point, with issues in non-reporting and selective reporting bias remaining inadequately addressed, which can hinder accurate treatment assessment and evidence synthesis.

Additionally, the study highlights the role of AI or LLMs in evaluating research quality. While AI platforms like ChatGPT and Gemini showed moderate agreement with human assessments, their potential for consistent and accurate evaluation still requires further refinement, particularly in the interpretation of complex data like graphs. AI’s speed and efficiency in evaluating large volumes of papers present a significant advantage, but inconsistencies in repeated evaluations and difficulties with visual data suggest that human expertise remains crucial in this process.

Moving forward, it is recommended that future updates to the PRISMA guidelines incorporate specific items regarding the role of AI in research evaluations to ensure transparency, consistency, and accuracy. AI tools have the potential to assist in large-scale evaluations, but they must be used with caution, and their limitations must be acknowledged. The study also underscores the importance of addressing the gaps in bias reporting, sensitivity analysis, and research registration, which remain critical areas for improvement in ophthalmology-related systematic reviews and meta-analyses. As AI technology continues to evolve, its integration into the research evaluation process will need careful consideration to maximise its potential while maintaining the integrity of human judgment in ensuring high-quality reporting. 

## Supplementary Information

Supplementary Material 1: Appendix 1.

## Data Availability

No datasets were generated or analysed during the current study.
