# Supplementary material for: Compliance of systematic reviews and meta-analyses in ophthalmology with the PRISMA statement: an AI-based assessment and longitudinal comparison with 2017 data
Source: BMC Med Res Methodol. 2026 Mar 17;26:92. doi: 10.1186/s12874-026-02825-0 (PMC13107902; doi:10.1186/s12874-026-02825-0)
Supplement: Supplementary file 1 — Supplementary Material 1: Appendix 1. [file 12874_2026_2825_MOESM1_ESM.docx]

**Appendix 1**

Ovid MEDLINE(R) ALL <1946 to May 01, 2025>

1 "systematic review"/ or systemic review.mp. 293179

2 meta analysis.mp. or meta analysis/ 338022

3 "progress in retinal and eye research".jn. 969

4 ophthalmology.jn. 17777

5 jama ophthalmology.jn. 4571

6 ocular surface.jn. 1364

7 "survey of ophthalmology".jn. 3314

8 "annual review of vision science".jn. 228

9 "clinical and experimental ophthalmology".jn. 4224

10 "contact lens and anterior eye".jn. 1801

11 "american journal of ophthalmology".jn. 28299

12 "british journal of ophthalmology".jn. 21876

13 "asia pacific journal of ophthalmology".jn. 419

14 1 or 2 481990

15 3 or 4 or 5 or 6 or 7 or 8 or 9 or 10 or 11 or 12 or 13 84842

16 14 and 15 888

17 limit 16 to yr="2020 - 2024" 351

18 limit 17 to english language 351

Embase <1974 to 2025 May 01>

1 "systematic review"/ or systemic review.mp. 543426

2 meta analysis.mp. or meta analysis/ 510511

3 "progress in retinal and eye research".jn. 1083

4 ophthalmology.jn. 18837

5 jama ophthalmology.jn. 4712

6 ocular surface.jn. 1456

7 "survey of ophthalmology".jn. 3298

8 "annual review of vision science".jn. 213

9 "clinical and experimental ophthalmology".jn. 7325

10 "contact lens and anterior eye".jn. 2723

11 "american journal of ophthalmology".jn. 20036

12 "british journal of ophthalmology".jn. 15548

13 "asia pacific journal of ophthalmology".jn. 514

14 1 or 2 792239

15 3 or 4 or 5 or 6 or 7 or 8 or 9 or 10 or 11 or 12 or 13 75745

16 14 and 15 1252

17 limit 16 to yr="2020 - 2024" 496

18 limit 17 to english language 496

* The indicated date refers to the day on which the search strategy and the article list were last cross-checked and verified.
